# Genetic and Functional Associations with Decreased Anti-inflammatory Tumor Necrosis Factor Alpha Induced Protein 3 in Macrophages from Subjects with Axial Spondyloarthritis

**DOI:** 10.3389/fimmu.2017.00860

**Published:** 2017-07-24

**Authors:** Yiping Liu, Zhan Ye, Xiang Li, Jennifer L. Anderson, Mike Khan, Douglas DaSilva, Marissa Baron, Deborah Wilson, Vera Bocoun, Lynn C. Ivacic, Steven J. Schrodi, Judith A. Smith

**Affiliations:** ^1^Department of Pediatrics, University of Wisconsin-Madison, Madison, WI, United States; ^2^Biomedical Informatics Research Center, Marshfield Clinic Research Institute, Marshfield, WI, United States; ^3^Integrated Research and Development Laboratory, Marshfield Clinic Research Institute, Marshfield, WI, United States; ^4^Department of Pathology and Laboratory Medicine, University of Wisconsin-Madison School of Medicine and Public Health, Madison, WI, United States; ^5^Graduate School of Public Health, University of Pittsburgh, Pittsburgh, PA, United States; ^6^Department of Rheumatology, Marshfield Clinic, Marshfield, WI, United States; ^7^Center for Human Genetics, Marshfield Clinic Research Institute, Marshfield, WI, United States; ^8^Computation and Informatics in Biology and Medicine, University of Wisconsin-Madison, Madison, WI, United States

**Keywords:** tumor necrosis factor alpha-induced protein 3, spondyloarthritis, cytokine production, macrophages, genetic variants, spondyloarthritis pathogenesis, TNF-α

## Abstract

**Objective:**

Tumor necrosis factor alpha-induced protein 3 (TNFAIP3) is an anti-inflammatory protein implicated in multiple autoimmune and rheumatologic conditions. We hypothesized that lower levels of TNFAIP3 contributes to excessive cytokine production in response to inflammatory stimuli in axial spondyloarthritis (AxSpA). A further aim was to determine the immune signaling and genetic variation regulating TNFAIP3 expression in individual subjects.

**Methods:**

Blood-derived macrophages from 50 AxSpA subjects and 30 healthy controls were assessed for TNFAIP3 expression. Cell lysates were also analyzed for NF-κB, mitogen-activated protein (MAP) kinase and STAT3 phosphorylation, and supernatants for cytokine production. Coding and regulatory regions in the *TNFAIP3* gene and other auto-inflammation-implicated genes were sequenced by next-generation sequencing and variants identified.

**Results:**

Mean TNFAIP3 was significantly lower in spondyloarthritis macrophages than controls (*p* = 0.0085). Spondyloarthritis subject macrophages correspondingly produced more TNF-α in response to lipopolysaccharide (LPS, *p* = 0.015). Subjects with the highest TNFAIP3 produced significantly less TNF-α in response to LPS (*p* = 0.0023). Within AxSpA subjects, those on TNF blockers or with shorter duration of disease expressed lower levels of TNFAIP3 (*p* = 0.0011 and 0.0030, respectively). TNFAIP3 expression correlated positively with phosphorylated IκBα, phosphorylated MAP kinases, and unstimulated phosphorylated STAT3, but negatively with LPS or TNF-α-stimulated fold induction of phosphorylated STAT3. Further, subjects with specific groups of variants within *TNFAIP3* displayed differences in TNFAIP3 (*p* = 0.03–0.004). Nominal pQTL associations with genetic variants outside *TNFAIP3* were identified.

**Conclusion:**

Our results suggest that both immune functional and genetic variations contribute to the regulation of TNFAIP3 levels in individual subjects. Decreased expression of TNFAIP3 in AxSpA macrophages correlated with increased LPS-induced TNF-α, and thus, TNFAIP3 dysregulation may be a contributor to excessive inflammatory responses in spondyloarthritis subjects.

## Introduction

Tumor necrosis factor alpha-induced protein 3 (TNFAIP3/A20) is an anti-inflammatory ubiquitin modifying enzyme implicated in multiple autoimmune and autoinflammatory disorders (1). Disease-associated single-nucleotide polymorphisms (SNPs) in the *TNFAIP3* gene have been identified in genome-wide association studies (GWAS) in systemic lupus erythematosus (SLE), rheumatoid arthritis (RA), type I diabetes, Crohn’s disease, celiac disease, primary biliary cirrhosis, systemic sclerosis, and psoriasis (1, 2). More recently, haploinsufficiency of TNFAIP3 has been described in six families with Behcet’s disease (3).

*TNFAIP3* encodes an 89.6-kDa cytosolic protein with a deubiquitinating OTU (ovarian tumor) domain, and seven zinc-finger domains containing ubiquitin-binding and E3 ubiquitin-ligating activities that modulate multiple proteins within inflammatory signaling cascades. The ubiquitinating region of TNFAIP3 attaches K48-linked polyubiquitin, thus targeting molecules such as receptor-interacting protein (RIP-1) or the E2 ubiquitin-conjugating protein UBCH5C for proteasome destruction. TNFAIP3 also removes K63-linked polyubiquitin chains required for the activity of molecules, such as RIP-1, TNFR-associated factor 6, and Tank-binding kinase (TBK1) upstream of NF-κB activation. TNFAIP3 thus antagonizes signaling downstream of pattern recognition receptors (PRRs) such as toll-like receptors (TLRs) and NOD-like receptors, as well as cytokine receptors [reviewed in Ref. (1)]. Ultimately, the net inhibition of these signaling molecules results in decreased activation of NF-κB family transcription factors, which are key regulators of inflammatory cytokines such as IL-6 and TNF-α. NF-κB signaling also induces TNFAIP3 expression, thus providing a further feedback loop that modifies inflammation.

Knockout mice have revealed the importance of TNFAIP3 in controlling systemic inflammation: knockout mice die shortly after birth from overwhelming multi-organ inflammation (4). A20/TNFAIP3 knockout mice are also exquisitely hypersensitive to TNF and lipopolysaccharide (LPS)-induced toxicity. The results of TNFAIP3-deficient unfettered inflammation appears dependent upon cell type and other unknown factors: mice lacking TNFAIP3 specifically in lysozyme M expressing macrophages and granulocytes develop an RA phenotype, whereas mice conditionally deficient for TNFAIP3 in CD11c dendritic cells either develop SLE or manifest more of a spondyloarthritis phenotype, with enthesitis, axial disease, and gut pathology (independent studies performed at different institutions) (5–8). CD11c-specific TNFAIP3-deficient mice produce excess IL-2, IL-10, IL-12, IFN-γ, and TNF-α in response to LPS (9).

Spondyloarthritis, including the prototypical ankylosing spondylitis (AS), encompasses a group of autoinflammatory rheumatologic conditions affecting 1–2% of Americans that incurs high rates of morbidity (10, 11). Due to the insidious progression of the disease and difficulty in correct diagnosis, irreversible damage often occurs prior to therapeutic intervention. An increased understanding of the pathogenesis of spondyloarthritis is critical for prompt diagnosis and may lead to efficacious, targeted therapies. Susceptibility to spondyloarthritis is clearly complex and heterogeneous, involving multiple genes and environmental insults (12–14). GWAS, in conjunction with studies in animal models and immunological studies in humans, have supported the etiological role of excessively activated, dysfunctional signaling in the IL-23/IL-17 pathway as a key driver of the disease (15, 16). However, the causal mechanisms behind the pathogenesis of spondyloarthritis remain enigmatic.

Interestingly, *TNFAIP3* has not emerged from AS-specific GWAS, even though it has been implicated in related diseases with clinical overlap, such as psoriasis and inflammatory bowel disease (IBD) (17, 18). To date, GWAS have only identified an estimated 25% of the heritability for AS (14, 19). The “missing heritability” may reflect unidentified rare variants, inherited epigenetic modifications, or functional interaction among identified genes. Alternatively, the expression or activity of disease-related immune molecules may be indirectly regulated by upstream molecules. For instance, the heritability of TNFAIP3 effects may be attributable to alleles segregating at genes responsible for post-transcriptional regulation (e.g., protein cleavage, phosphorylation, ubiquitination, and/or glycosylation) (20–22). Despite the absence of *TNFAIP3*-linked SNPs in AS GWAS, our previous study of gene expression in macrophages from AS subjects revealed lower levels of *TNFAIP3* message (57% of control) (23). Another study has shown decreased TNFAIP3 mRNA in peripheral blood mononuclear cells (PBMCs) of AS patients (24). Furthermore, studies of *in vitro*-stimulated macrophages from AS subjects have revealed increased pro-inflammatory cytokine production in response to infectious stimuli, such as PRR agonists (25). Although multiple factors converge to regulate inflammatory cytokine production, we hypothesized that low TNFAIP3 may contribute to excessive cytokine production in spondyloarthritis. In this study, we undertook a detailed examination of a population of individuals with axial spondyloarthritis (AxSpA) and healthy controls to compare TNFAIP3 levels and determine genetic and functional factors associated with TNFAIP3 variation.

## Materials and Methods

### Subjects

Fifty patients with diagnosis codes for AS (ICD-9 code 720.0) or spondyloarthritis (721.9) were recruited equally from the University of Wisconsin-Madison Rheumatology clinics and the Marshfield Clinic and Research Foundation. Axial disease was documented by X-ray or MRI and confirmed with their primary rheumatologists. Sacroiliitis grade was determined by radiology report according to modified New York criteria. Thirty healthy controls with no personal or family history of AS, personal history of RA, intercurrent infection, or history of cancer requiring immunomodulatory therapy, were also recruited equally from the same institutions. Subjects gave their written consent prior to peripheral venipuncture. The study was reviewed and approved by the Marshfield Clinic Institutional Review Board.

### Blood DNA Processing and Next-Generation Targeted Sequencing (NGS)

Peripheral blood was collected using 10 ml purple top EDTA tubes for DNA purification (Becton-Dickinson). Genomic DNA was extracted using Gentra Puregene Blood Kit (QIAGEN) protocol. Library preparation was performed using a custom-designed HaloPlex (Agilent Technologies) enrichment kit targeting 48 selected regions based on 28 genes from the literature involved in AS, psoriasis, and IBD susceptibility (26–30). All exons and introns were covered ≥86% in the probe selection process. Additionally, putative transcription factor-binding sites or other regulatory elements containing cis-eQTL polymorphisms (as determined by the web-based software Genevar from the Wellcome Trust Sanger Institute at *p* < 0.05 in HapMap CEU samples) adjacent (both upstream and downstream) to those genes were selected (31). Refinement of targeted regions was performed using the SureDesign (Agilent Technologies) software. All samples were run concurrently on the MiSeq (Illumina) system, thereby avoiding batch effects. Six sequencing runs, using V3 600 and 150 cycle kits on a single pooled library preparation, provided sufficient read depth and quality. All binary sequence alignment/map files (BAM files^1^) generated on the sequencing runs were analyzed together to call sequence variants using the Genome Analysis ToolKit software package (GATK version 3.2-2) following the GATK Best Practices recommendations (32, 33). ANNOVAR was employed to annotate called variants (34). HLA-B27 status was determined by an established TaqMan (ThermoFisher) genotyping assay interrogating rs4349859. For QC on the genotyping assay, 100% concordance with 10 CEU HapMap samples was noted (5 reference allele homozygous samples and 5 heterozygous samples) and the results on the case and control DNAs exhibited excellent discrimination performance to call genotypes.

### Macrophage Derivation

40 mL of blood was collected in yellow top acid citrate dextrose tubes (BD). The blood was centrifuged 10 min at 4°C and plasma removed. An equal amount of PBS (Corning) was added to the cell layer and the mixture transferred to polystyrene tubes (Fisher Scientific) containing LSM™ Lymphocyte Separation Medium (MP Biomedicals). Tubes were centrifuged 15 min at room temperature. PBMCs were collected with a disposable Pasteur pipet (Fisher Scientific), washed twice with buffer [PBS with 2% fetal bovine serum (FBS) and 2 mM EDTA]. Human monocytes were purified using the human monocyte isolation kit II (Miltenyi Biotec) according to the manufacturer’s instructions. Purified monocytes were plated in RPMI 1.5–2 h and then non-adherent cells removed. Adherent cells were subsequently incubated in complete RPMI 1640 medium (RPMI 1640 with 10% FBS (HyClone), 4 mM l-glutamine, 100 U/mL penicillin, 100 µg/mL streptomycin) supplemented with 20 ng/mL recombinant human M-CSF (PeproTech) on days 0, 2, and 4 post-plating. Macrophage function and protein expression were further assessed on day 6 from cultures initially set up in parallel. In previous experience, this approach results in a homogeneous appearing, CD163+ population of cells with morphologic features of macrophages (25).

### Functional Assays

#### Cytosolic Protein Expression

Cells were lysed in Bio-Rad lysis buffer, sonicated 30 s, and stored at −80°C until analysis. TNFAIP3 and CARD9 levels were quantified by ELISA (MyBioSource) with normalization to lysate protein concentration as determined by bicinchoninic assay (Thermo Scientific).

#### Cytokine Supernatant Tests

On day 5 post isolation, cells were stimulated with 10 ng/mL LPS, 100 nM PGE2, or both for 24 h. Supernatants were assayed for IL-23 and TNF-α concentration by ELISA (eBioscience) and IL-6, IL-8, and IL-12p70 by Luminex (eBioscience). After supernatants were removed, the cells were lysed in 100 µL type lysis buffer for determination of cellular protein concentration as a cell number surrogate.

#### Cell Signaling

Macrophages were stimulated with 10 ng/mL LPS, 1 ng/mL TNF-α, 100 µg/mL curdlan, or 200 ng/mL IL-23 for 30 min and then cells were lysed in 1× lysis buffer (Bio-Rad luminex kit). pATF2, pJNK, p38, pNF-κB p65, pERK, and pSTAT3 were analyzed using a multiplex luminex assay (Bio-rad Pro Phosphoprotein magnetic 6-plex) and pIκBα was assayed using the MILLIPLEX MAP Phospho IκBα (Ser32) Magnetic Bead MAPmate (Millipore). Phosphoproteins were detected on a Luminex 100 system machine. Cytokine and cell signaling results were all normalized to cellular protein concentration. One AxSpA biochemistry sample had much lower protein amounts (<10% on average compared to other samples) and was discarded from the correlational analysis of signaling events.

### Statistics

#### Analysis of Protein Levels

A Shapiro–Wilk *W* test was used to test protein expression data for normality. Significant departures from a Gaussian model for the protein-adjusted TNFAIP3 ratios were treated by normalizing *via* a Box–Cox transformation, better enabling the use of linear regression approaches. Comparisons between mean protein expression levels in dichotomous outcomes were made using a *T*-statistic and 1 M permutations to obtain a *p*-value. To test correlation patterns for continuous outcomes, Spearman’s rs correlation coefficients were calculated and corresponding *p*-values testing departure from rs = 0 are presented. To test for clustering patterns in TNFAIP3 protein levels across AxSpA samples in an exploratory fashion, both *K*-means (varying *k*) and UPGMA clustering methods were applied to examine the data visually for separation between AxSpA cases. Experiment-wise significance levels were determined using a Bonferroni approach.

#### Analysis of Genetic Data

For the purposes of quality control, an exact test of Hardy–Weinberg equilibrium (HWE) was applied to genotype data at each variant and performed separately for cases and controls. Variants exhibiting HWE *p* < 1E−05 were considered potentially problematic and excluded from analyses of the endpoints. To test the null hypothesis of independence between genotypes at each variant and case/control status, a logistic regression model was constructed under additivity of genetic effects, adjusted for age and sex covariates. HWE, and logistic and linear regression analyses were performed in the R programming language using existing statistical routines. Haplotype analyses for variants segregating at the *TNFAIP3* gene region utilized a Gibbs sampling algorithm to estimate phase. Pairwise linkage disequilibrium calculations were performed using the NCI LDlink tool and comparisons were made to the CEU and GRB HapMap sample sets using *r*^2^. To test for differences in the distributions of variants in dichotomous outcomes, a Kolmogorov–Smirnov test was used. Experiment-wise significance levels were determined using a Bonferroni approach.

#### pQTL Analysis

To test the null hypothesis of independence between TNFAIP3/total protein values and genetic variants, a linear regression model was used, also adjusted for age and sex. These pQTL analyses were performed separately for cases, controls, and the combined group of all individuals. For computational efficiency, both the logistic regression and linear regression models used asymptotic null distributions for the calculation of *p*-values. To verify the *p*-values generated for the top 30 findings, a permutation routine was run to obtain permuted *p*-values under the same model.

#### Permutation Routine

The permutation routine was written in the XLISP-STAT programming language to test the null hypothesis of exchangeability between case/control statuses in analyses of dichotomous outcomes. In each permutation, the case/control status variable was randomized by assignment to a uniformly distributed random variable and sorted. A *T*-statistic was calculated for each permuted iteration and compared to the *T*-statistic calculated for the observed data. A permuted *p*-value was obtained by calculating the frequency of permuted runs that exceeded the observed *T*-statistic. Given that the permuted *p*-value is binomially distributed, one million iterations was deemed sufficient in producing highly stable *p*-value estimates for these data given that the 95% confidence interval for a *p*-value of 0.0001 is (0.00008, 0.00012), for a *p*-value of 0.001 is (0.00094, 0.0011), and for a *p*-value of 0.01 is (0.0098, 0.0102). For the test of TNFAIP3/protein levels vs. AxSpA/Control endpoint, we also calculated the Mann–Whitney *U* test for the purposes of comparison with the permuted results.

## Results

Following the ASAS classification criteria introduced in 2009, “ankylosing spondylitis” was subsumed under the classification rubric of “axial spondyloarthritis” (35). This broader group of AxSpA was studied herein (characteristics in Table 1). The percentage of HLA-B27 positive subjects and related odds ratio (82) was comparable to previous studies of AxSpA (19, 36). Although a standard instrument was not used to document severity or disease activity, provider notes suggested most patients had mild or inactive disease activity (29/46) with only 37% having “moderate” or “severe” activity. Extra-articular manifestations included 18 (36%) with history of uveitis, 4 (8%) with psoriatic rash, and 2 (4%) with IBD, comparable to reports in AS and non-radiographic AxSpA (37, 38). Regarding medications in the AxSpA subjects, 27 (54%) were on TNF blockers, 28 (56%) on NSAIDs with 12 (24%) NSAIDs only, 10 (20%) on DMARDs (5 on sulfasalazine and 5 on methotrexate), 4 (8%) on prednisone, and 4 on no medications. Median age of onset was 26.5 years.

**Table 1:** Subject characteristics.

Characteristic	Axial SpA (50)	Controls (30)	*p* Values
Age (mean ± SD in years)	52 ± 13	51 ± 11	0.71
Sex %M (number)	56 (28/50)	37 (11/30)	0.10
Race/ethnicity % and (number Non-Caucasian)	0	7 (2/30)	NS
HLA-B27 positive % (number)	84 (42/50)	7 (2/30)	3 × 10^−12^
Family history ankylosing spondylitis (AS)% (number)	34 (17/50)	0	
Disease duration (mean ± SD, years)	21 ± 12	NA	
Extra-articular disease% (number)^a^	40 (20/50)	NA	
On TNF blockers% (number)^b^	54 (27/50)	NA	

*All axial SpA subjects had MRI or X-ray documented spinal disease and carried a diagnosis of either spondyloarthritis or AS, ascertained by diagnostic code and confirmed by their primary rheumatologists*.

*^a^This includes 18 with uveitis, 4 with psoriatic rash, and 2 with inflammatory bowel disease*.

*^b^Numbers taking TNF-blocking therapy at the time of sample collection. This includes 17 on antibody type medication (infliximab, adalimumab, golimumab) and 10 on TNF soluble receptors (etanercept, certolizumab pegol)*.

We focused on blood-derived macrophages for multiple reasons: macrophages are abundant in histologic sections from inflamed enthesitis and sacroiliac joints in AS (39, 40). In articular and gut specimens from AS patients, myeloid cells produce the IL-23 implicated in pathogenesis (41, 42). Macrophages from AS patients secrete excess TNF-α and IL-23 in response to LPS (25). Finally, macrophages express many of the molecules implicated in AS GWAS (26). Cultured differentiated macrophages were utilized rather than blood monocytes to mitigate effects of on-going systemic inflammation or circulating therapeutics. TLR agonist-induced cytokine production from these cultured macrophages is highly reproducible in independent experiments over time (Figure S1 in Supplementary Material).

### Lower TNFAIP3 Levels in AxSpA Patients

**Figure 1:**
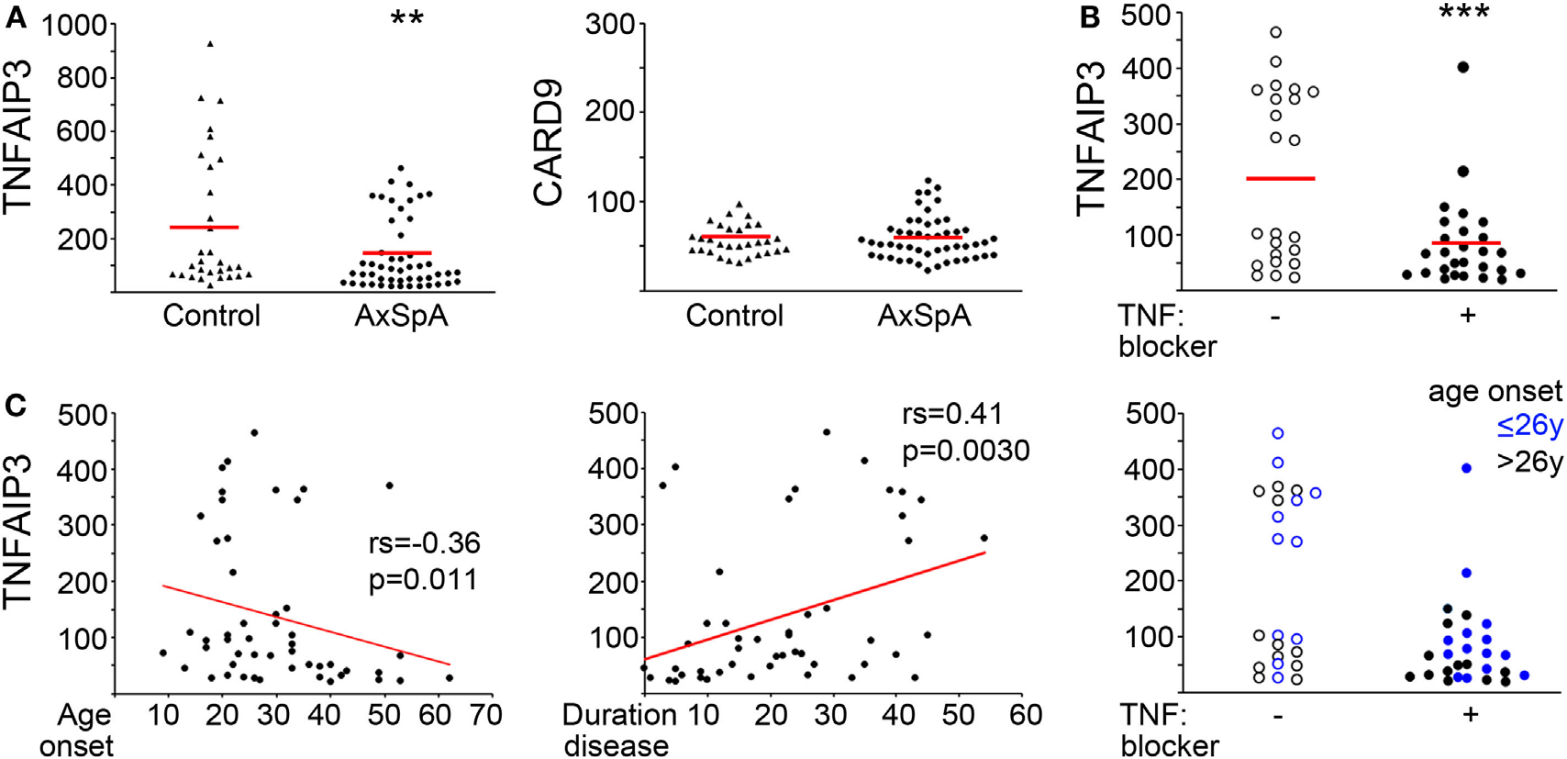
Decreased tumor necrosis factor alpha-induced protein 3 (TNFAIP3) levels in axial spondyloarthritis (AxSpA) subjects. **(A)** Peripheral blood-derived macrophage lysates were assessed for TNFAIP3 or CARD9 protein by ELISA. Healthy controls are denoted by triangles and AxSpA subjects by circles. The red lines designate mean expression. **T-test statistic with 1,000,000 (1 M) permutations *p* = 0.0085. AxSpA patient sample TNFAIP3 levels were further analyzed according to **(B)** current TNF blocker use (open circles for no TNF blocker and filled circles for current TNF blocker). **(C)** age of onset (left, years), duration of disease (middle, years) or both age of onset and TNF blocker status (right). ***p = 0.0011 by permuted *T*-test. Significance for age of onset and duration of disease were determined by Spearman’s correlation.

Although there was overlap between controls and AxSpA patients, over 40% of the AxSpA patients expressed TNFAIP3 below the control 25th percentile and none above the control 75th percentile. Thus there was a markedly skewed distribution in AxSpA subjects, with over representation of low TNFAIP3 values and no patients with elevated levels. Mean expression for AxSpA TNFAIP3 was 138.0 (100, 175 95% CI) vs. 255.4 (157, 353) (permuted *p* = 0.0085; Mann–Whitney *U* test *p* = 0.019) for control and median AxSpA TNFAIP3 was 77.8 vs. 113 in controls. By contrast, in the same lysates, CARD9 expression did not differ significantly and exhibited similar distributions (Figure 1). Within the AxSpA subjects, there appeared to be 2 populations, those below and above 200, which was substantiated by a clustering analysis. TNFAIP3 level did not correlate with sex, HLA-B27 status, family history of AS or reported disease activity, but did correlate with age of onset (rs = −0.36, *p* = 0.01) and disease duration (rs = 0.41, *p* = 0.0030). Subjects on TNF blockers or those with disease onset after the median of age 26 almost exclusively had lower TNFAIP3 levels. There were no significant differences in TNFAIP3 levels between those on antibody type anti-TNF medication (e.g., infliximab, adalimumab) and those taking TNF soluble receptors such as etanercept [average 91.7 (44,140) vs. 70.2 (36, 105), respectively]. Thus, analysis by TNF blocker, age of onset, and TNFAIP3 levels revealed at least three distinct subgroups within “axial spondyloarthritis.”

### Functional Correlations with TNFAIP3 Levels

Tumor necrosis factor alpha-induced protein 3 would be predicted to impact PRR and cytokine-receptor-mediated biochemical signaling, for instance inhibiting downstream NF-κB and mitogen-activated protein (MAP) kinase activation (9, 43). Activity of these same pathways have also been implicated in the induction of TNFAIP3 itself (diagram in Figure S2 in Supplementary Material) (1, 44). To determine the relationship between TNFAIP3 levels and inflammatory signaling and cytokine production in our subject macrophages, we examined the NF-κB pathway (p65, pIκBα), MAP kinase signaling (p38, pERK, pJNK, downstream pATF2) and STAT3 phosphorylation (pSTAT3) in response to cytokine, and PRR agonist stimuli. Culture supernatants were also assessed for cytokine production.

**Figure 2:**
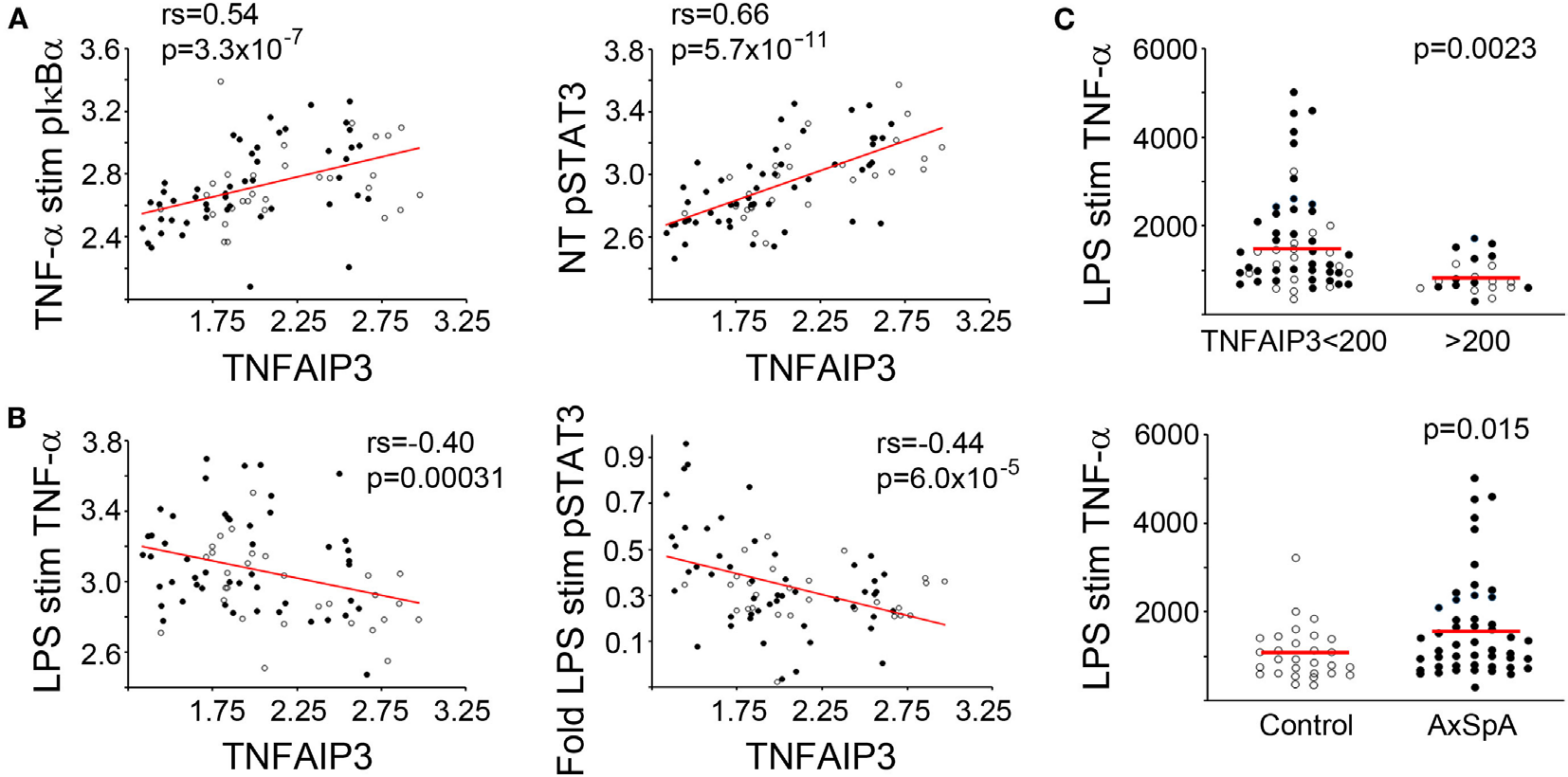
Functional correlations with tumor necrosis factor alpha-induced protein 3 (TNFAIP3) levels. Blood-derived macrophages were stimulated with media (no treatment or NT), TNF-α, or lipopolysaccharide (LPS). For biochemical signaling studies, cells were lysed after 30 min of stimulation and amount of phosphorylated signaling molecule determined by Luminex assay. For cytokine determination, cells were stimulated for 24 h and supernatant levels of TNF-α quantified using ELISA. Cytokine production and phosphorylated molecules were normalized by total protein. Pairwise correlations (Spearman’s correlation coefficient) between TNFAIP3 levels and signaling/cytokine output and corresponding *p*-values were determined (see Table 2). Axial spondyloarthritis (AxSpA) subjects are depicted with filled black circles and controls with open circles. In panels **(A,B)**, red lines show correlations for log-transformed data. **(A)** Positive correlations with TNFAIP3 levels. **(B)** Negative correlations with TNFAIP3 levels. In panel **(C)**, red lines denote mean TNF-α production in each group. LPS-induced TNF-α production separated by TNFAIP3 level (top, *p* = 0.0023) or spondyloarthritis status (bottom, *p* = 0.015) with *p*-values from a *T*-test statistic with 1 M permutations.

Tumor necrosis factor alpha-induced protein 3 expression correlated with specific functional outcomes, both upstream and downstream of TNFAIP3 (Table 2; with examples in Figure 2). We observed highly significant positive correlations between phosphorylated IkBα (Figure 2A), MAP kinase (e.g., ERK, JNK) and TNFAIP3 levels. These particular results also suggest that at least some of the regulation of TNFAIP3 is likely to be related to factors outside the *TNFAIP3* gene itself in the cultured macrophages. Regarding events downstream of TNFAIP3, we observed correlations with increased PGE2-induced IL-6, but decreased LPS-stimulated TNF-α (*r* = −0.32, *p* = 0.00031, Figure 2B). Indeed in subjects with TNFAIP3 levels >200, LPS-induced TNF production was significantly lower [mean 863 (689, 1037) vs. 1622 (1329, 1919), *p* = 0.0024, Figure 2C]. Just as AxSpA subjects had lower TNFAIP3 levels than controls, LPS-induced TNF-α production was greater in AxSpA subjects than controls [mean 1618 (1286, 1950) vs. 1053 (824, 1282), *p* = 0.016]. There was also a non-significant trend for macrophages from subjects on TNF blockers (who had lower TNFAIP3) to produce greater TNF-α in response to LPS as compared to those not on TNF blockers (mean 1822 vs. 1386, *p* = 0.19). TNFAIP3 levels also negatively correlated with IL-12p70 production (*p* < 2 × 10^−5^), though IL-12 was only detected at low levels (<30 pg/mL, data not shown). The correlation between TNFAIP3 and pNF-κB/other cytokine induction was weaker (Rho between −0.3 and −0.4) but generally in the negative direction. For instance, the correlation between TNFAIP3 and LPS-induced fold increase in phospho-p65 (NF-κB) was −0.36 (*p* = 0.001), and TNFAIP3 and LPS-induced fold increase in IL-6 was −0.33 (*p* = 0.003). The negative correlation between TNFAIP3 and LPS-induced cytokine production, are consistent with known suppression of LPS and TNF-α signaling by TNFAIP3 (4). In comparison to p65 and cytokine induction, fold induction of ERK was also positive (*r* = 0.38, *p* = 0.00072 for LPS pERK vs. NT). The correlations with STAT3 were more complex, in that TNFAIP3 correlated with elevated pSTAT3 under certain conditions [e.g., baseline NT (Figure 2A)], but negatively correlated with fold induction in response to LPS (Figure 2B) and TNF-α stimulation.

**Table 2:** Functional correlations with tumor necrosis factor alpha-induced protein 3 protein levels in macrophages.

Stimulus	output	Spearman’s rho	*p*-Value
**Curdlan**	**pSTAT3**	**0.66**	**5.1 × 10^−11^**
**NT**	**pSTAT3**	**0.66**	**5.7 × 10^−11^**
**IL-23**	**pSTAT3**	**0.63**	**5.42 × 10^−10^**
**Curdlan**	**pJNK**	**0.52**	**9.0 × 10^−7^**
**TNF-α**	**pIκBα**	**0.54**	**3.3 × 10^−7^**
**Untreated**	**pIκBα**	**0.51**	**3.2 × 10^−6^**
LPS	pIκBα	0.48	8.8 × 10^−6^
IL-23	pIκBα	0.47	4.6 × 10**^−^**^5^
**Curdlan**	**pERK1/2**	**0.49**	**6.0 × 10^−6^**
LPS	pERK1/2	0.43	9.9 × 10^−5^
Curdlan	fold pERK	0.43	7.3 × 10^−5^
**Curdlan**	**pATF2**	**0.49**	**4.3 × 10^−6^**
LPS	pATF2	0.41	2.3 × 10^−4^
PGE2	IL-6	0.44	3.8 × 10^−5^
LPS	fold pSTAT3	−0.44	6.0 × 10^−5^
TNF-α	fold pSTAT3	−0.43	7.5 × 10^−5^

*Blood-derived macrophages were stimulated with media (no treatment or NT), Curdlan (dectin-1 agonist), LPS, TNF-α, or IL-23 for 30 min prior to lysis for biochemical analyses by multiplex Luminex bead assays. For cytokine studies, cells were stimulated for 24 h with PGE2, LPS, or both. Supernatants were assessed for TNF-α, IL-23p19, IL-12p70, IL-6, and IL-8 by Luminex or ELISA. Fold induction is the ratio vs. untreated. Only correlations with rho ≥0.4 (moderate correlations) and *p* ≤ 0.001 are presented. Associations with *p* < 8 × 10^−6^ (bold) retain significance after Bonferroni correction. Positive correlations are listed above negative correlations*.

### TNFAIP3 Genetic Analyses

**Figure 3:**
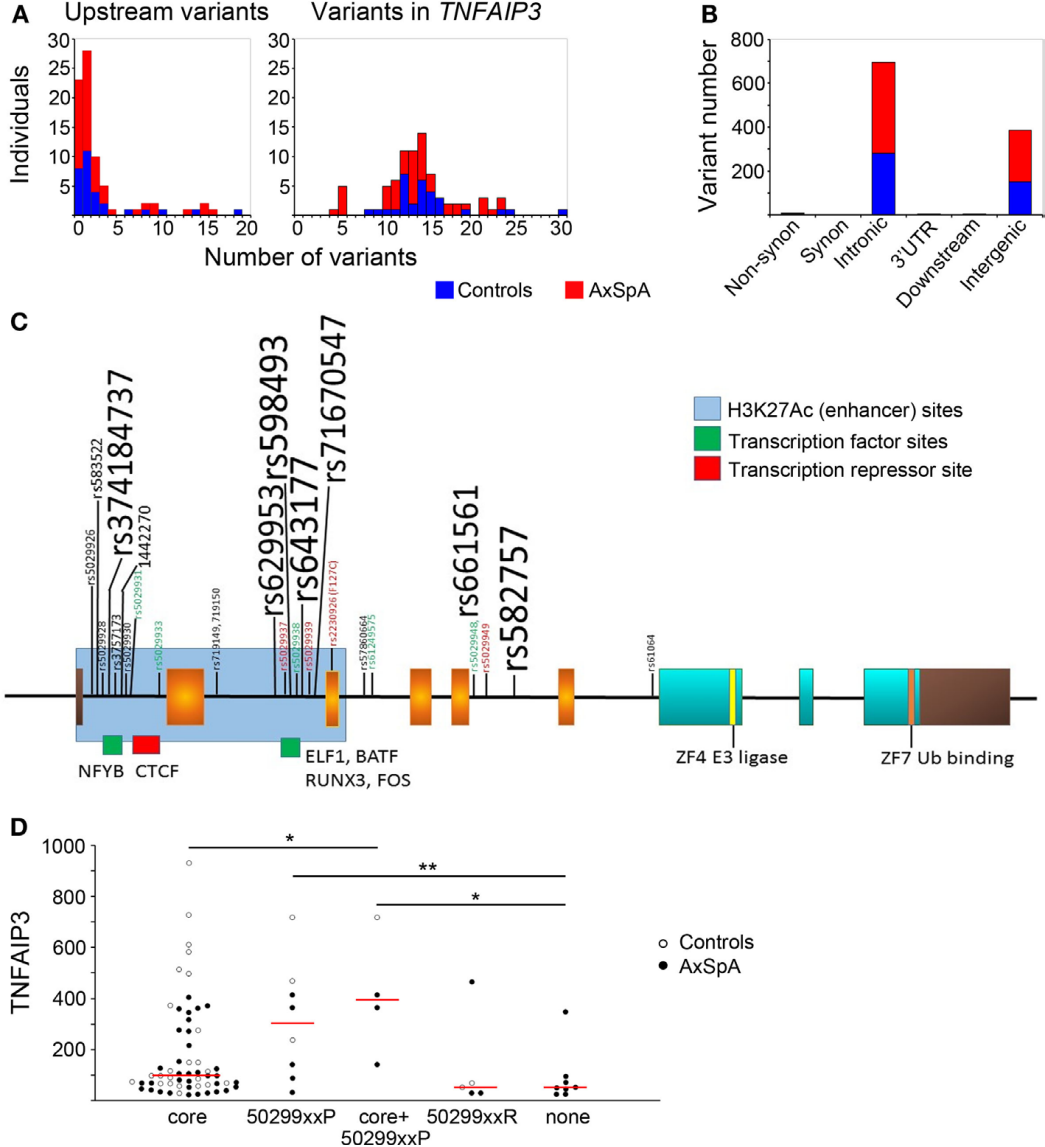
Distribution of variants within and upstream of *TNFAIP3* and association with expression. *TNFAIP3* exons and introns as well as putative regulatory regions (including the upstream promoter region) were sequenced by next-generation sequencing. **(A)** Numbers of axial spondyloarthritis (AxSpA) subjects (red bars) and healthy controls (blue bars) with specified numbers of upstream (left) or *TNFAIP3* variants (right). **(B)** Distribution of variants within the *TNFAIP3* gene according to functional region. **(C)** Variant map: exons encoding the OTU domain are in orange, zinc fingers in aqua and non-coding regions are brown. Approximate sites of H3K27Ac histone marks (blue region) and transcription factor-binding sites (green and red) are as described at http://genome.ucsc.edu. Location of rs-numbered variants present in at least 4 of 80 subjects are depicted. Variant font sizes correlate with number of subjects with the variant. Subjects were assessed as “low” expressers if ≤25th percentile of controls. Red font refers to variants where at least 2/3 subjects had low tumor necrosis factor alpha-induced protein 3 (TNFAIP3) expression and green font to <17% of subjects having low TNFAIP3 expression. **(D)** The groups of variants include subjects with common “core” variants, those with “protective” variants (“50299xxP”) associated with more moderate-high expression, a third group with 502xxP and core variants, those with “risk” variants (“50299xxR”) associated with lower expression, and a last group with none of these variants (“none”). One individual with both protective and risk variants was excluded from the analysis related to potentially conflicting effects. AxSpA subjects are in filled circles and controls in open circles. Red lines denote median expression in variant groups (*p ≤ 0.05, **p ≤ 0.005).

In addition to functional regulation *via* PRR/cytokine signaling, GWAS suggest genetic regulation of TNFAIP3. Thus, we did a detailed analysis of the *TNFAIP3* gene region by NGS, tallying variants present in all individuals and correlating them with protein level. More variants were generally present in the gene itself (Figure 3A) than upstream (mean 3 ± 4 variants in both cases and in controls with most having 0–1 variants upstream). Within the *TNFAIP3* gene region, subjects had an average of 14 ± 5 variants with most being intronic or intergenic (Figure 3B). SpA cases and controls had the same number of intronic (mean eight to nine per subject) and intergenic (mean five per subject) variants. There were nine subjects with single non-synonymous coding variants, six of nine being the previously described F127C variant (rs2230926), and the others T647P, P587L, and G744D of unclear significance.

Within *TNFAIP3*, introns 1 and 2 were most heavily hit by genetic variation (Figure 3C). Interestingly, these regions have enhancer marks and transcription-binding sites per the UCSC genome browser.^2^ The most common variants in our subjects did not have any discernable effect on distribution of TNFAIP3 protein levels. Some of the less common variants were associated with skewed levels of TNFAIP3, as indicated by colored font in Figure 3. In particular, intronic variants rs5029928, rs5029931, rs5029933, rs5029938, rs5029948, rs61259575 (all p_permuted ≤0.04), and intergenic variant rs73566259 (*p* = 0.029) rarely occurred in subjects with low TNFAIP3. These intronic variants displayed high linkage disequilibrium. Two tightly linked variants, rs719149 and rs719150, associated with lower levels of TNFAIP3 in AxSpA cases only but not controls (*p* = 0.016 for SpA status). Variants rs5029937, rs5029939, and F127C have previously been reported to associate with RA and SLE and showed a trend toward lower levels in this study (45, 46). However, none of the individual variants identified were statistically significant following correction for multiple variant testing. An analysis was performed comparing case and control variant number in putative functional regions (regulatory motifs, non-synonymous substitutions, proximal untranslated regions). The mean genetic risk score for these variants within axial SpA was 6.04 with SD of 4.66. The mean within controls was 5.48 with a SD of 5.46. The permuted *T*-test (1 M iterations) yielded a *p*-value of 0.641 and Kolmogorov–Smirnov test of distributional differences gave a *p*-value of 0.09 (no significant difference).

Upon examination of the linkage disequilibrium structure among individual *TNFAIP3* variants present in our samples, we noted interesting patterns (Figure 3D). There was a “core” set of common variants (rs3741847, rs629953, rs598493, rs643177, rs7167054, rs661561, rs582757) slightly more frequent in controls than AxSpA (83–100 vs. 76–88%, not significant) associated with a wide distribution of TNFAIP3 levels. There were also sets of protective variants (“50299xxP”) associated with higher TNFAIP3, such as rs5029931, 5029933, and 5029948. Sets of risk variants (“50299xxR”), including rs5029937 and rs5029939, tended to associate with lower TNFAIP3 protein. Subjects without any of these variants also had lower TNFAIP3 levels (*p* = 0.03 and 0.004, Figure 3D). In a haplotype analysis, only this last group significantly associated with lower TNFAIP3 levels and this haplotype was more common in AxSpA cases than controls (Cochran–Armitage *p*-value of 0.043).

### Positional Association of *PTPN2* Region with TNFAIP3 Levels

**Figure 4:**
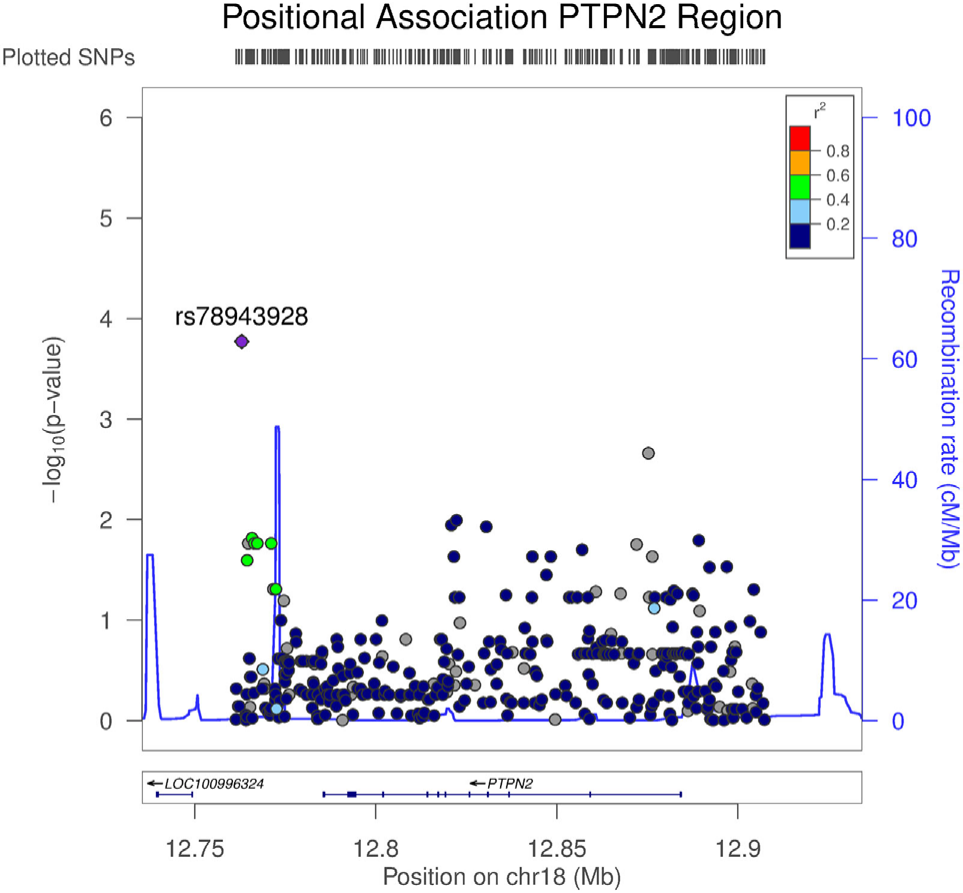
Association of variants in PTPN2 with tumor necrosis factor alpha-induced protein 3 (TNFAIP3) protein expression in axial spondyloarthritis (AxSpA) subjects. The region covering *PTPN2* spanned approximately 146 kbp. Variants were determined from targeted next-generation sequencing (NGS) and the high-quality variants used in this analysis totaled 364. *p* Values testing the correlation of each genetic variant in the *PTPN2* region with normalized TNFAIP3 levels within the AxSpA subjects were generated by LocusZoom (http://csg.sph.umich.edu/locuszoom/). The statistical approach used a linear regression model adjusted for age and sex. Linkage disequilibrium (*r*^2^) between the variant exhibiting the highest level of significance and the linked variants is denoted by color. Additionally, estimated recombination rate across the region is shown.

To assess associations between TNFAIP3 protein levels and genetic variants outside *TNFAIP3*, a pQTL analysis was performed on the other autoimmunity/spondyloarthropathy implicated genes selected for NGS (Table S1 in Supplementary Material). To further investigate the normalized TNFAIP3 pQTL effects, we focused on the 50 AxSpA samples and evaluated all high-quality variants. For this analysis of AxSpA-only data, 5,065 variants passed the high-quality criteria. Within the *PTPN2* targeted region, 364 high-quality variants were identified. The results from the linear regression model, adjusted for age and sex covariates, yielded several nominally significant variants in the *PTPN2* region including the top finding of rs78943928 (*p* = 1.69E−04) which resides approximately 29 and 22 kbp 3′ of *PTPN2* isoform 1 and isoform 2, respectively. Several additional variants in this targeted region, such as the *PTPN2* intronic variants rs592390, rs2542162, and rs12971201, also exhibited nominally significant correlation with normalized TNFAIP3 levels (Table S1 in Supplementary Material). Figure 4 shows the positional association results for the *PTPN2*-linked variants. The G allele (minor allele) at rs78943928, the minor allele (frequency = 0.0688 in all samples), was positively correlated with increased normalized TNFAIP3 levels in AxSpA subjects. The allele frequency was consistent with that observed in CEU samples (freq *G* = 0.058) and the phase 3 1000 Genomes European sample set (freq *G* = 0.0726). Neither AxSpA samples nor controls exhibited a significant departure from HWE.

## Discussion

Inflammatory cytokines have long been known to play a critical role in fomenting arthritis and spondyloarthritis. Indeed, the seminal work by Sherlock et al. showing the sufficiency of over-expressed IL-23 to yield an enthesitis/ankylosis phenotype in a mouse model testifies to the centrality of cytokines in spondyloarthritis (47). Other mouse models of soluble (TNFΔARE) or membrane bound TNF-α overexpression also result in enthesitis and spinal arthritis/ankylosis phenotypes (48, 49). Abundant TNF-α-producing cells have been described in early sacroiliitis lesions from AS patients (50). In a previous study, macrophages from AS subjects (more narrowly defined than “AxSpA” in this study) overproduced IL-23 and TNF-α in response to LPS (25). If overproduction of pro-inflammatory cytokines is central to disease pathogenesis, and possibly initiating spondyloarthritis, the mechanisms underlying overproduction have remained unclear. The MHC allele HLA-B27 may increase cytokine responses to PRR agonists *via* the pro-inflammatory cellular stress response known as the unfolded protein response, but not all spondyloarthritis patients are HLA-B27 positive (51, 52). In this study, we found that monocyte-derived macrophages from AxSpA subjects expressed less of the anti-inflammatory protein TNFAIP3 than control subjects. Conversely, AxSpA macrophages produced greater levels of TNF-α in response to LPS. Given the relationship we observed between elevated TNFAIP3 and decreased TNF-α, the lower levels of TNFAIP3 in AxSpA subjects may contribute to excessive cytokine production in response to PRR agonists. The degree of correlation between TNFAIP3 and PRR-induced cytokine in this study may reflect contributions from other inputs (e.g., CD14 and TLR4 variants, other downstream signaling molecules).

Tumor necrosis factor alpha-induced protein 3 expression was significantly lower in AxSpA subjects with higher age of onset, shorter disease duration, and on TNF blockers. Interestingly, RA subjects with lower TNFAIP3 have been reported to have greater response to anti-TNF medications (53). In subjects who are not treatment naïve, there is always a concern for artifact related to medication. However, it is not obvious from a mechanistic standpoint how previous cellular exposure to TNF blockers would result in greater cytokine production, particularly after a washout period during sample processing and 6-day *in vitro* culture. Also, there was no difference between anti-TNF antibodies, which might remain cell-surface bound and soluble TNF “sinks.” One hypothesis is that these subjects had more inflammatory disease, thus meriting treatment with TNF blockers. In our study, reported clinical activity and radiologic assessment did not support a direct relationship between disease severity and TNFAIP3 level (Figure S3 in Supplementary Material). However, macrophages from subjects on TNF blockers tended to produce higher levels of TNF-α, though numbers were not sufficient to detect a significant difference. Similarly, another study by Gibellini et al. reported that PBMC from psoriatic responders to adalimumab and etanercept produced greater levels of TNF-α *in vitro* (54). The same study by Gibellini et al. also reported a change in monocyte phenotype for those responding to anti-TNF agents, with an increase in classical CD14+ monocytes (vs. CD16+ monocytes). On the other hand, anti-TNF therapy decreased percentages of CD56+ pro-inflammatory monocytes in RA (55). Although the net effect of TNF blockade is unclear, the possibility exists that our starting population of monocytes may be subtly different between subjects on or off TNF blockers. The reason for the association of lower TNFAIP3 with older age of onset is also unclear. We did note a correlation between TNFAIP3 and duration of disease, so those with longer standing disease and ankylosis may be in a more “burned out,” less inflammatory stage of disease. Together, these results suggest the presence of multiple immune/clinical subgroups present under the heading of “AxSpA.”

Upon investigation of the regulation of TNFAIP3 in all of our subjects together, we found both functional and genetic correlates with TNFAIP3 protein levels. TNFAIP3 levels displayed moderately strong positive correlations with pIκBα as well as multiple phosphorylated MAP kinases. Our results are consistent with the known induction of TNFAIP3 by such signals (1, 44). These results and the negative correlation with cytokine induction provide validation for our TNFAIP3 quantification. One question that arises is how to put together these positive and negative functional correlations with disease status. Our study was not generally powered to detect biochemical signaling differences between AxSpA disease subjects and controls, particularly given the apparent heterogeneity in arthritis subjects. However, one hypothesis is as follows: in subjects with higher TNFAIP3 (controls >subjects in this study), there is a greater tonic (or baseline) activation of NF-κB and MAP kinase phosphorylation that is balanced by the induction of anti-inflammatory TNFAIP3. In arthritis subjects, decreased basal levels of TNFAIP3 results in greater fold induction of NF-κB upon stimulation. Perhaps, it is the dynamic fold induction of NF-κB signaling that is more important for PRR stimulation of inflammatory cytokines than tonic or absolute level of pathway activation. The positive correlation between TNFAIP3 levels and fold induction of pERK suggest TNFAIP3 may regulate NF-κB and MAP kinase signaling differently. Relief of tonic inflammatory stimulation may be one reason why subjects on TNF blockers had less TNFAIP3 but does not explain the existence of two discrete groups (high and low) among subjects who were not on TNF blockers. For a diagrammatic summary of findings and hypothesis, see Figure S4 in Supplementary Material.

The strongest positive and negative functional correlations for TNFAIP3 levels were with pSTAT3. Indeed, these correlations were even more significant than for pIκBa and p65 (NF-κB) regulation. GWAS have identified *STAT3* as a susceptibility gene in both Crohn’s disease and AS, the latter in both Europeans and Han Chinese (56, 57). Thus, the finding of an association between TNFAIP3 and STAT3 activation may have implications for disease pathogenesis. In our study, higher TNFAIP3 associated with greater pSTAT3, particularly in untreated or weakly stimulated *in vitro* conditions. However, higher TNFAIP3 also correlated with decreased fold induction of pSTAT3 in response to LPS and TNF-α stimulation. At this time, very little information is available regarding the interaction of TNFAIP3 and STAT3 activation, and what is there appears disease/tissue dependent: in liver, TNFAIP3 activity results in increased STAT3 phosphorylation and IL-6 induction *via* inhibition of suppressor of cytokine signaling 3 (58). Yet myeloid-specific A20 deficiency is associated with elevated IL-6-dependent RA in a mouse model (5). In this study, our results suggest that lower TNFAIP3 may contribute to a greater fold induction of pSTAT3 in response to inflammatory stimuli such as TNF-α and LPS. The STAT3 transcription factor critically regulates the development of Th17 cells and directly binds the promoters of the *IL21* and *IL17* genes (59). Thus, exaggerated pSTAT3 fold induction could enhance the IL-23/IL-17 pathway, a cytokine pathway now clearly implicated in AS pathogenesis (15).

In this study, we provided a detailed snapshot of genetic variation within the *TNFAIP3* gene at the individual level in groups of AxSpA and controls. Our analysis of *TNFAIP3* indicated that most of the variation lies in the intronic regions of *TNFAIP3* rather than promoter or coding regions, with similar distributions between cases and controls. In particular, introns 1 and 2 seem most heavily inundated with variation. At first glance, one might think introns would be less subject to purifying selection given the severity of phenotype in knockout mice. However, these two introns appear to be locations of enhancer histone marks and transcription factor consensus-binding sites. Thus, variation in this region might be expected to provide more subtle differences in gene regulation than, for instance, a promoter variant.

Some of the *TNFAIP3* variants found in our population have been reported in other diseases: rs5029937 and F127C with RA and SLE, rs5029939 with SLE and systemic sclerosis, and F127C and rs610604 with psoriasis (45, 46, 60, 61). However, despite the increasing implication of TNFAIP3 in multiple autoimmune and inflammatory diseases, little information is available regarding how *TNFAIP3* variants modulate gene expression. Rs610604 has previously been associated with lower mRNA expression in the setting of coronary artery disease and type 2 diabetes (62). F127C (rs2230926), located within the deubiquitinating region, renders TNFAIP3 modestly less effective at inhibiting TNF-α-induced NF-κB signaling, and one study associates rs2230926 TT homozygosity with lower mRNA levels and poorer outcome in RA (60, 63). In our study, the subjects with these previously described disease-risk variants exhibited a non-significant trend toward having lower TNFAIP3 levels (4/6 subjects at or below the control 25%). The association of alleles with higher TNFAIP3 levels has not been well described: there is only one report of a T-cell leukemia line bearing the rs5029948 variant with high TNFAIP3 expression level (64). Our numbers were too small to perform highly powered eQTL analyses, and the nominal association values found in this study should be followed up. In general, our study was also not powered to discern differences in specific variant frequency between cases and controls. However, since an AxSpA GWAS has not been published, moving forwards, heterogeneity may be as much of an issue as sample size. The issue with genetic heterogeneity in “axial spondyloarthritis” has been highlighted recently as genetic scores failed to predict disease any better than clinical assessment (65). Although individual *TNFAIP3* variants were not highly significant in this study, interestingly, TNFAIP3 levels differed according to groups of variants. In particular, the subjects with referent allele haplotypes had the lowest expression and subjects carrying groups of “protective” alleles had higher expression (Figure 3D). Only the referent *TNFAIP3* sequence, more common in AxSpA, exhibited statistically significant results in the haplotype analysis. These results will also require confirmation in a much larger cohort.

Outside of the *TNFAIP3* gene, the top finding for the TNFAIP3 pQTL analysis of high-quality NGS variant data within AxSpA cases was rs78943928, a SNP residing within 30 kbp 3′ of the protein tyrosine phosphatase non-receptor-type encoding gene, *PTPN2*. This region immediately downstream of PTPN2 has been strongly implicated by GWAS in a number of systemic inflammatory diseases including psoriasis and IBD (17, 66). Within the *PTPN2* coding region, studies have demonstrated additional association findings with RA and celiac disease (67). Interestingly, rs78943928 was not in substantial linkage disequilibrium with any of the various SNPs found to be GWAS significant in these other disease studies. Studies have clearly shown that ptpn2 regulates both adaptive and innate immunity and loss of this molecule results in a hyperinflammatory state (68): Ptpn2−/− mice overproduce cytokines, develop arthritis, and die young of systemic inflammatory disease (69). Recently, Wiede and colleagues have shown that reduction of ptpn2 expression increases autoreactivity of CD8+ T-cells (70). Given the considerable overlap between clinical features, molecular pathogenesis and susceptibility loci across psoriasis, Crohn’s disease, and AS, the observation of *PTPN2* genetic signal for the TNFAIP3 pQTL analysis in AxSpA may provide additional insight into AxSpA etiology and warrants further investigation.

In summary, although much has been learned about how TNFAIP3 shapes immune responses in mice, and GWAS have picked up multiple TNFAIP3 SNP disease associations, very little is known regarding how TNFAIP3 is regulated or how it shapes immune function within human subjects. In particular, it has been unclear how individual SNPs relate to each other or to TNFAIP3 protein levels. In this study, we sought to bring together genetics and immune function in a synthetic approach to elucidate TNFAIP3 regulation in AxSpA. Herein, we found decreased levels of the anti-inflammatory protein TNFAIP3 in M-CSF-derived macrophages from subjects with AxSpA, constituting a potential contributor to cytokine dysregulation in this arthritic disease. More specifically, based on functional correlations, the lower levels of TNFAIP3 in AxSpA subjects may predispose toward increased fold induction of pSTAT3, p65 NF-κB, and TNF-α in response to PRR agonists such as LPS. Our novel open-ended approach to variant discovery *via* next-generation sequencing has allowed us to map *TNFAIP3* variation across coding, intronic, and regulatory regions in this cohort to an unprecedented extent, elucidating relative abundance of variants and their association with TNFAIP3 levels. Finally, this open approach has enabled discovery of pQTL variants outside the *TNFAIP3* gene itself (e.g., *PTPN2*), including a SNP not identified by other GWAS.

Although GWAS have pointed to associations between genetic variants and disease, the genetic architecture within individuals that conspires to produce disease remains mysterious. Along these lines, we are only beginning to scratch the surface of how these genetic variants orchestrate subtle changes in immune function within individuals. This type of knowledge may only come about through detailed functional and genetic analysis at the individual subject level, as performed here, but in large numbers of subjects. Analyses of individual genes will also have to be integrated into more global genetic, epigenetic, and functional networks to provide insight into net immune function.

## Ethics Statement

The study was carried out in accordance with the ethical principles outlined in the Belmont report and Common Rule, with written informed consent from all subjects. All subjects gave written informed consent in accordance with the Declaration of Helsinki. The protocol was approved by the Marshfield Clinic Institutional Review Board.

## Author Contributions

All authors listed have read the manuscript except XL. She has returned to China without leaving a forwarding email address and is not reachable. YL—conducting experiments and manuscript writing; ZY and XL—data analysis; JA—data analysis and manuscript writing; MK—conducting experiments; DD—experimental design, data acquisition, and conducting experiments; MB—conducting experiments; DW and VB—data acquisition; LI—conducting experiments; SS and JS—funding, experimental design, data analysis, and manuscript writing.

## Conflict of Interest Statement

The authors declare that the research was conducted in the absence of any commercial or financial relationships that could be construed as a potential conflict of interest.

## Footnotes

^1^http://samtools.sourceforge.net/.

^2^www.genome.ucsc.edu.

## Supplementary Material

Table S1 Association of genetic variants outside TNFAIP3 with TNFAIP3 protein level. (A) Case–Control Results: all case–control association results across all targeted regions that exceeded alpha = 0.05 in significance for high quality variants are presented. 50 Cases and 30 Controls were used in the analysis. A logistic regression model, adjusted for age and sex, was used to generate the p-values. Permutation results showed strong consistency with the asymptotic p-values. The information in the table includes: targeted gene region (Gene Region), reference and alternative alleles at the high quality variant (Ref/Alt), the basic positional annotation (Annotation), the rs number for the variant (SNP), chromosome (Chr), genomic position (hg19/dbSNP build 147), p-value for an exact test of Hardy–Weinberg equilibrium in cases (HWExact_P_case), p-value for an exact test of Hardy–Weinberg equilibrium in controls (HWExact_P_control), and finally, the case–control association p-value (Case–Control Adj p-value). (B,C) TNFAIP3 pQTL All Samples: (B) shows the results from the pQTL analysis performed on normalized TNFAIP3 protein levels, using cases and controls (B) or AxSpA cases only (C). The results are restricted to high quality variants obtained from targeted gene regions and exceeding alpha = 0.05 in statistical significance. Association p-values are from the adjusted linear regression model (TNFAIP3/protein ALL p-value).

## References

[1] Ma A Malynn BA. A20: linking a complex regulator of ubiquitylation to immunity and human disease. Nat Rev Immunol (2012) 12(11):774–85. 10.1038/nri3313 23059429 PMC3582397

[2] Cordell HJ Han Y Mells GF Li Y Hirschfield GM Greene CS International genome-wide meta-analysis identifies new primary biliary cirrhosis risk loci and targetable pathogenic pathways. Nat Commun (2015) 6:8019. 10.1038/ncomms9019 26394269 PMC4580981

[3] Zhou Q Wang H Schwartz DM Stoffels M Park YH Zhang Y Loss-of-function mutations in TNFAIP3 leading to A20 haploinsufficiency cause an early-onset autoinflammatory disease. Nat Genet (2016) 48(1):67–73. 10.1038/ng.3459 26642243 PMC4777523

[4] Lee EG Boone DL Chai S Libby SL Chien M Lodolce JP Failure to regulate TNF-induced NF-kappaB and cell death responses in A20-deficient mice. Science (2000) 289(5488):2350–4. 10.1126/science.289.5488.2350 11009421 PMC3582399

[5] Matmati M Jacques P Maelfait J Verheugen E Kool M Sze M A20 (TNFAIP3) deficiency in myeloid cells triggers erosive polyarthritis resembling rheumatoid arthritis. Nat Genet (2011) 43(9):908–12. 10.1038/ng.874 21841782

[6] Kool M van Loo G Waelput W De Prijck S Muskens F Sze M The ubiquitin-editing protein A20 prevents dendritic cell activation, recognition of apoptotic cells, and systemic autoimmunity. Immunity (2011) 35(1):82–96. 10.1016/j.immuni.2011.05.013 21723156

[7] Hammer GE Turer EE Taylor KE Fang CJ Advincula R Oshima S Expression of A20 by dendritic cells preserves immune homeostasis and prevents colitis and spondyloarthritis. Nat Immunol (2011) 12(12):1184–93. 10.1038/ni.2135 22019834 PMC3419270

[8] De Wilde K Martens A Lambrecht S Jacques P Drennan MB Debusschere K A20 inhibition of STAT1 expression in myeloid cells: a novel endogenous regulatory mechanism preventing development of enthesitis. Ann Rheum Dis (2016) 76(3):585–92. 10.1136/annrheumdis-2016-209454 27551052

[9] Xuan NT Wang X Nishanth G Waisman A Borucki K Isermann B A20 expression in dendritic cells protects mice from LPS-induced mortality. Eur J Immunol (2015) 45(3):818–28. 10.1002/eji.201444795 25472594

[10] Sieper J Braun J Rudwaleit M Boonen A Zink A. Ankylosing spondylitis: an overview. Ann Rheum Dis (2002) 61(Suppl 3):iii8. 10.1136/ard.61.suppl_3.iii8 12381506 PMC1766729

[11] Boonen A Severens JL. Ankylosing spondylitis: what is the cost to society, and can it be reduced? Best Pract Res Clin Rheumatol (2002) 16(4):691–705. 10.1016/S1521-6942(02)90244-5 12406435

[12] Pedersen OB Svendsen AJ Ejstrup L Skytthe A Harris JR Junker P Ankylosing spondylitis in Danish and Norwegian twins: occurrence and the relative importance of genetic vs. environmental effectors in disease causation. Scand J Rheumatol (2008) 37(2):120–6. 10.1080/03009740701824613 18415769

[13] Brown MA Kennedy LG MacGregor AJ Darke C Duncan E Shatford JL Susceptibility to ankylosing spondylitis in twins: the role of genes, HLA, and the environment. Arthritis Rheum (1997) 40(10):1823–8. 10.1002/art.1780401015 9336417

[14] Brown MA Kenna T Wordsworth BP Genetics of ankylosing spondylitis – insights into pathogenesis. Nat Rev Rheumatol (2016) 12(2):81–91. 10.1038/nrrheum.2015.133 26439405

[15] Smith JA Colbert RA The IL-23/IL-17 axis in spondyloarthritis pathogenesis: Th17 and beyond. Arthritis Rheum (2013) 66(2):231–41. 10.1002/art.38291 PMC4058712 24504793

[16] Gaston JS Goodall JC Baeten D Interleukin-23: a central cytokine in the pathogenesis of spondylarthritis. Arthritis Rheum (2011) 63(12):3668–71. 10.1002/art.30600 22127689

[17] Wellcome Trust Case Control Consortium. Genome-wide association study of 14,000 cases of seven common diseases and 3,000 shared controls. Nature (2007) 447(7145):661–78. 10.1038/nature05911 17554300 PMC2719288

[18] Nair RP Duffin KC Helms C Ding J Stuart PE Goldgar D Genome-wide scan reveals association of psoriasis with IL-23 and NF-kappaB pathways. Nat Genet (2009) 41(2):199–204. 10.1038/ng.311 19169254 PMC2745122

[19] Reveille JD Genetics of spondyloarthritis – beyond the MHC. Nat Rev Rheumatol (2012) 8(5):296–304. 10.1038/nrrheum.2012.41 22487796

[20] Coornaert B Baens M Heyninck K Bekaert T Haegman M Staal J T cell antigen receptor stimulation induces MALT1 paracaspase-mediated cleavage of the NF-kappaB inhibitor A20. Nat Immunol (2008) 9(3):263–71. 10.1038/ni1561 18223652

[21] Hutti JE Turk BE Asara JM Ma A Cantley LC Abbott DW. IkappaB kinase beta phosphorylates the K63 deubiquitinase A20 to cause feedback inhibition of the NF-kappaB pathway. Mol Cell Biol (2007) 27(21):7451–61. 10.1128/MCB.01101-07 17709380 PMC2169042

[22] Shrikhande GV Scali ST da Silva CG Damrauer SM Csizmadia E Putheti P O-glycosylation regulates ubiquitination and degradation of the anti-inflammatory protein A20 to accelerate atherosclerosis in diabetic ApoE-null mice. PLoS One (2010) 5(12):e14240. 10.1371/journal.pone.0014240 21151899 PMC2997780

[23] Smith JA Barnes MD Hong D DeLay ML Inman RD Colbert RA. Gene expression analysis of macrophages derived from ankylosing spondylitis patients reveals interferon-gamma dysregulation. Arthritis Rheum (2008) 58(6):1640–9. 10.1002/art.23512 18512784 PMC2888278

[24] Duan R Leo P Bradbury L Brown MA Thomas G. Gene expression profiling reveals a downregulation in immune-associated genes in patients with AS. Ann Rheum Dis (2010) 69(9):1724–9. 10.1136/ard.2009.111690 19643760

[25] Zeng L Lindstrom MJ Smith JA. Ankylosing spondylitis macrophage production of higher levels of interleukin-23 in response to lipopolysaccharide without induction of a significant unfolded protein response. Arthritis Rheum (2011) 63(12):3807–17. 10.1002/art.30593 22127699 PMC3228355

[26] International Genetics of Ankylosing Spondylitis Consortium (IGAS) Cortes A Hadler J Pointon JP Robinson PC Karaderi T Identification of multiple risk variants for ankylosing spondylitis through high-density genotyping of immune-related loci. Nat Genet (2013) 45(7):730–8. 10.1038/ng.2667 23749187 PMC3757343

[27] Ellinghaus D Jostins L Spain SL Cortes A Bethune J Han B Analysis of five chronic inflammatory diseases identifies 27 new associations and highlights disease-specific patterns at shared loci. Nat Genet (2016) 48(5):510–8. 10.1038/ng.3528 26974007 PMC4848113

[28] Białecka M Ostasz R Kurzawski M Klimowicz A Fabiańczyk H Bojko P IL17A and IL17F gene polymorphism association with psoriasis risk and response to treatment in a Polish population. Dermatology (2016) 232(5):592–6. 10.1159/000448090 27591988

[29] Pointon JJ Chapman K Harvey D Sims AM Bradbury L Laiho K Toll-like receptor 4 and CD14 polymorphisms in ankylosing spondylitis: evidence of a weak association in Finns. J Rheumatol (2008) 35(8):1609–12. 18634146

[30] Australo-Anglo-American Spondyloarthritis Consortium (TASC) Reveille JD Sims AM Danoy P Evans DM Leo P Genome-wide association study of ankylosing spondylitis identifies non-MHC susceptibility loci. Nat Genet (2010) 42(2):123–7. 10.1038/ng.513 20062062 PMC3224997

[31] Yang TP Beazley C Montgomery SB Dimas AS Gutierrez-Arcelus M Stranger BE Genevar: a database and Java application for the analysis and visualization of SNP-gene associations in eQTL studies. Bioinformatics (2010) 26(19):2474–6. 10.1093/bioinformatics/btq452 20702402 PMC2944204

[32] McKenna A Hanna M Banks E Sivachenko A Cibulskis K Kernytsky A The genome analysis toolkit: a MapReduce framework for analyzing next-generation DNA sequencing data. Genome Res (2010) 20(9):1297–303. 10.1101/gr.107524.110 20644199 PMC2928508

[33] Van der Auwera GA Carneiro MO Hartl C Poplin R Del Angel G Levy-Moonshine A From FastQ data to high confidence variant calls: the genome analysis toolkit best practices pipeline. Curr Protoc Bioinformatics (2013) 43:11.10.1–33. 10.1002/0471250953.bi1110s43 25431634 PMC4243306

[34] Wang K Li M Hakonarson H. ANNOVAR: functional annotation of genetic variants from high-throughput sequencing data. Nucleic Acids Res (2010) 38(16):e164. 10.1093/nar/gkq603 20601685 PMC2938201

[35] Rudwaleit M van der Heijde D Landewé R Listing J Akkoc N Brandt J The development of assessment of spondyloarthritis international society classification criteria for axial spondyloarthritis (part II): validation and final selection. Ann Rheum Dis (2009) 68(6):777–83. 10.1136/ard.2009.108233 19297344

[36] Tournadre A Pereira B Lhoste A Dubost JJ Ristori JM Claudepierre P Differences between women and men with recent-onset axial spondyloarthritis: results from a prospective multicenter French cohort. Arthritis Care Res (Hoboken) (2013) 65(9):1482–9. 10.1002/acr.22001 23463610

[37] de Winter JJ van Mens LJ van der Heijde D Landewé R Baeten DL. Prevalence of peripheral and extra-articular disease in ankylosing spondylitis versus non-radiographic axial spondyloarthritis: a meta-analysis. Arthritis Res Ther (2016) 18:196. 10.1186/s13075-016-1093-z 27586785 PMC5009714

[38] Stolwijk C Essers I van Tubergen A Boonen A Bazelier MT De Bruin ML The epidemiology of extra-articular manifestations in ankylosing spondylitis: a population-based matched cohort study. Ann Rheum Dis (2015) 74(7):1378–8. 10.1136/annrheumdis-2014-205253 24658834

[39] Bollow M Fischer T Reisshauer H Backhaus M Sieper J Hamm B Quantitative analyses of sacroiliac biopsies in spondyloarthropathies: T cells and macrophages predominate in early and active sacroiliitis – cellularity correlates with the degree of enhancement detected by magnetic resonance imaging. Ann Rheum Dis (2000) 59(2):135–40. 10.1136/ard.59.2.135 10666170 PMC1753076

[40] McGonagle D Marzo-Ortega H O’Connor P Gibbon W Hawkey P Henshaw K Histological assessment of the early enthesitis lesion in spondyloarthropathy. Ann Rheum Dis (2002) 61(6):534–7. 10.1136/ard.61.6.534 12006328 PMC1754106

[41] Ciccia F Bombardieri M Principato A Giardina A Tripodo C Porcasi R Overexpression of interleukin-23, but not interleukin-17, as an immunologic signature of subclinical intestinal inflammation in ankylosing spondylitis. Arthritis Rheum (2009) 60(4):955–65. 10.1002/art.24389 19333939

[42] Appel H Maier R Bleil J Hempfing A Loddenkemper C Schlichting U In situ analysis of interleukin-23- and interleukin-12-positive cells in the spine of patients with ankylosing spondylitis. Arthritis Rheum (2013) 65(6):1522–9. 10.1002/art.37937 23508523

[43] Luo H Liu Y Li Q Liao L Sun R Liu X A20 regulates IL-1-induced tolerant production of CXC chemokines in human mesangial cells via inhibition of MAPK signaling. Sci Rep (2015) 5:18007. 10.1038/srep18007 26648169 PMC4673611

[44] Lai TY Wu SD Tsai MH Chuang EY Chuang LL Hsu LC Transcription of Tnfaip3 is regulated by NF-kappaB and p38 via C/EBPbeta in activated macrophages. PLoS One (2013) 8(9):e73153 10.1371/journal.pone.0073153 24023826 PMC3759409

[45] Zhang MY Yang XK Pan HF Ye DQ. Associations between TNFAIP3 gene polymorphisms and systemic lupus erythematosus risk: an updated meta-analysis. HLA (2016) 88(5):245–52. 10.1111/tan.12908 27726311

[46] Orozco G Hinks A Eyre S Ke X Gibbons LJ Bowes J Combined effects of three independent SNPs greatly increase the risk estimate for RA at 6q23. Hum Mol Genet (2009) 18(14):2693–9. 10.1093/hmg/ddp193 19417005 PMC2701332

[47] Sherlock JP Joyce-Shaikh B Turner SP Chao CC Sathe M Grein J IL-23 induces spondyloarthropathy by acting on ROR-gammat+ CD3+CD4-CD8- entheseal resident T cells. Nat Med (2012) 18(7):1069–76. 10.1038/nm.2817 22772566

[48] Kontoyiannis D Pasparakis M Pizarro TT Cominelli F Kollias G. Impaired on/off regulation of TNF biosynthesis in mice lacking TNF AU-rich elements: implications for joint and gut-associated immunopathologies. Immunity (1999) 10(3):387–98. 10.1016/S1074-7613(00)80038-2 10204494

[49] Edwards CK III Bendele AM Reznikov LI Fantuzzi G Chlipala ES Li L Soluble human p55 and p75 tumor necrosis factor receptors reverse spontaneous arthritis in transgenic mice expressing transmembrane tumor necrosis factor alpha. Arthritis Rheum (2006) 54(9):2872–85. 10.1002/art.22077 16947419

[50] François RJ Neure L Sieper J Braun J. Immunohistological examination of open sacroiliac biopsies of patients with ankylosing spondylitis: detection of tumour necrosis factor alpha in two patients with early disease and transforming growth factor beta in three more advanced cases. Ann Rheum Dis (2006) 65(6):713–20. 10.1136/ard.2005.037465 16249231 PMC1798185

[51] Turner MJ Sowders DP DeLay ML Mohapatra R Bai S Smith JA HLA-B27 misfolding in transgenic rats is associated with activation of the unfolded protein response. J Immunol (2005) 175(4):2438–48. 10.4049/jimmunol.175.4.2438 16081815

[52] Bowness P Hla-B27. Annu Rev Immunol (2015) 33:29–48. 10.1146/annurev-immunol-032414-112110 25861975

[53] Koczan D Drynda S Hecker M Drynda A Guthke R Kekow J Molecular discrimination of responders and nonresponders to anti-TNF alpha therapy in rheumatoid arthritis by etanercept. Arthritis Res Ther (2008) 10(3):R50. 10.1186/ar2419 18454843 PMC2483439

[54] Gibellini L Biasi SD Bianchini E Bartolomeo R Fabiano A Manfredini M Anti-TNF-alpha drugs differently affect the TNFalpha-sTNFR system and monocyte subsets in patients with psoriasis. PLoS One (2016) 11(12):e0167757 10.1371/journal.pone.0167757 27936119 PMC5147951

[55] Krasselt M Baerwald C Wagner U Rossol M. CD56+ monocytes have a dysregulated cytokine response to lipopolysaccharide and accumulate in rheumatoid arthritis and immunosenescence. Arthritis Res Ther (2013) 15(5):R139. 10.1186/ar4321 24286519 PMC3978677

[56] Danoy P Pryce K Hadler J Bradbury LA Farrar C Pointon J Association of variants at 1q32 and STAT3 with ankylosing spondylitis suggests genetic overlap with Crohn’s disease. PLoS Genet (2010) 6(12):e1001195. 10.1371/journal.pgen.1001195 21152001 PMC2996314

[57] Davidson SI Liu Y Danoy PA Wu X Thomas GP Jiang L Association of STAT3 and TNFRSF1A with ankylosing spondylitis in Han Chinese. Ann Rheum Dis (2011) 70(2):289–92. 10.1136/ard.2010.133322 21068102

[58] da Silva CG Studer P Skroch M Mahiou J Minussi DC Peterson CR A20 promotes liver regeneration by decreasing SOCS3 expression to enhance IL-6/STAT3 proliferative signals. Hepatology (2013) 57(5):2014–25. 10.1002/hep.26197 23238769 PMC3626749

[59] Korn T Bettelli E Oukka M Kuchroo VK. IL-17 and Th17 cells. Annu Rev Immunol (2009) 27:485–517. 10.1146/annurev.immunol.021908.132710 19132915

[60] Musone SL Taylor KE Lu TT Nititham J Ferreira RC Ortmann W Multiple polymorphisms in the TNFAIP3 region are independently associated with systemic lupus erythematosus. Nat Genet (2008) 40(9):1062–4. 10.1038/ng.202 19165919 PMC3897246

[61] Dieudé P Guedj M Wipff J Ruiz B Riemekasten G Matucci-Cerinic M Association of the TNFAIP3 rs5029939 variant with systemic sclerosis in the European Caucasian population. Ann Rheum Dis (2010) 69(11):1958–64. 10.1136/ard.2009.127928 20511617

[62] Boonyasrisawat W Eberle D Bacci S Zhang YY Nolan D Gervino EV Tag polymorphisms at the A20 (TNFAIP3) locus are associated with lower gene expression and increased risk of coronary artery disease in type 2 diabetes. Diabetes (2007) 56(2):499–505. 10.2337/db06-0946 17259397

[63] Zhu L Wang L Wang X Zhou L Liao Z Xu L Characteristics of A20 gene polymorphisms and clinical significance in patients with rheumatoid arthritis. J Transl Med (2015) 13:215. 10.1186/s12967-015-0566-1 26143186 PMC4491428

[64] Zhu L Zhang F Shen Q Chen S Wang X Wang L Characteristics of A20 gene polymorphisms in T-cell acute lymphocytic leukemia. Hematology (2014) 19(8):448–54. 10.1179/1607845414Y.0000000160 24611736

[65] Thomas GP Willner D Robinson PC Cortes A Duan R Rudwaleit M Genetic diagnostic profiling in axial spondyloarthritis: a real world study. Clin Exp Rheumatol (2017) 35(2):229–33. 27749235

[66] Baurecht H Hotze M Brand S Büning C Cormican P Corvin A Genome-wide comparative analysis of atopic dermatitis and psoriasis gives insight into opposing genetic mechanisms. Am J Hum Genet (2015) 96(1):104–20. 10.1016/j.ajhg.2014.12.004 25574825 PMC4289690

[67] Zhernakova A Stahl EA Trynka G Raychaudhuri S Festen EA Franke L Meta-analysis of genome-wide association studies in celiac disease and rheumatoid arthritis identifies fourteen non-HLA shared loci. PLoS Genet (2011) 7(2):e1002004. 10.1371/journal.pgen.1002004 21383967 PMC3044685

[68] Spalinger MR McCole DF Rogler G Scharl M. Protein tyrosine phosphatase non-receptor type 2 and inflammatory bowel disease. World J Gastroenterol (2016) 22(3):1034–44. 10.3748/wjg.v22.i3.1034 26811645 PMC4716018

[69] Doody KM Bussières-Marmen S Li A Paquet M Henderson JE Tremblay ML. T cell protein tyrosine phosphatase deficiency results in spontaneous synovitis and subchondral bone resorption in mice. Arthritis Rheum (2012) 64(3):752–61. 10.1002/art.33399 21968903

[70] Wiede F Ziegler A Zehn D Tiganis T PTPN2 restrains CD8(+) T cell responses after antigen cross-presentation for the maintenance of peripheral tolerance in mice. J Autoimmun (2014) 53:105–14. 10.1016/j.jaut.2014.05.008 24997008

