# Supplementary material for: Genetic and Functional Associations with Decreased Anti-inflammatory Tumor Necrosis Factor Alpha Induced Protein 3 in Macrophages from Subjects with Axial Spondyloarthritis
Source: Front Immunol. 2017 Jul 24;8:860. doi: 10.3389/fimmu.2017.00860 (PMC5523649; doi:10.3389/fimmu.2017.00860)
Supplement: Supplementary file 2 [file Image_1.pdf]

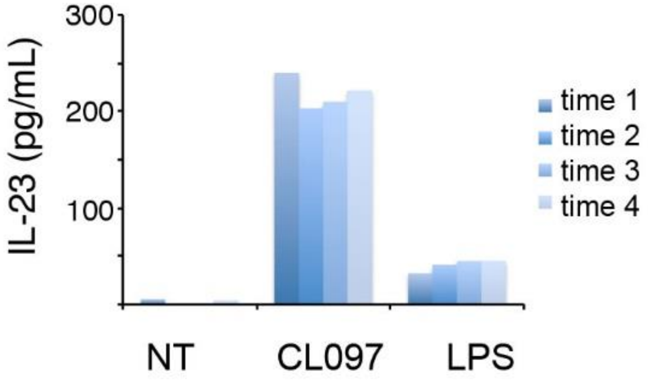

Figure S1. Reproducibility of cytokine production from blood-derived cultured macrophages. Peripheral blood derived macrophages from one individual were obtained >1 week apart on 4 separate occasions (times 1-4) and stimulated with media, CL097 (water-soluble TLR7 agonist) or Lipopolysaccharide (LPS). ELISA quantitated IL-23 is depicted with varying shades for different cellular isolations.

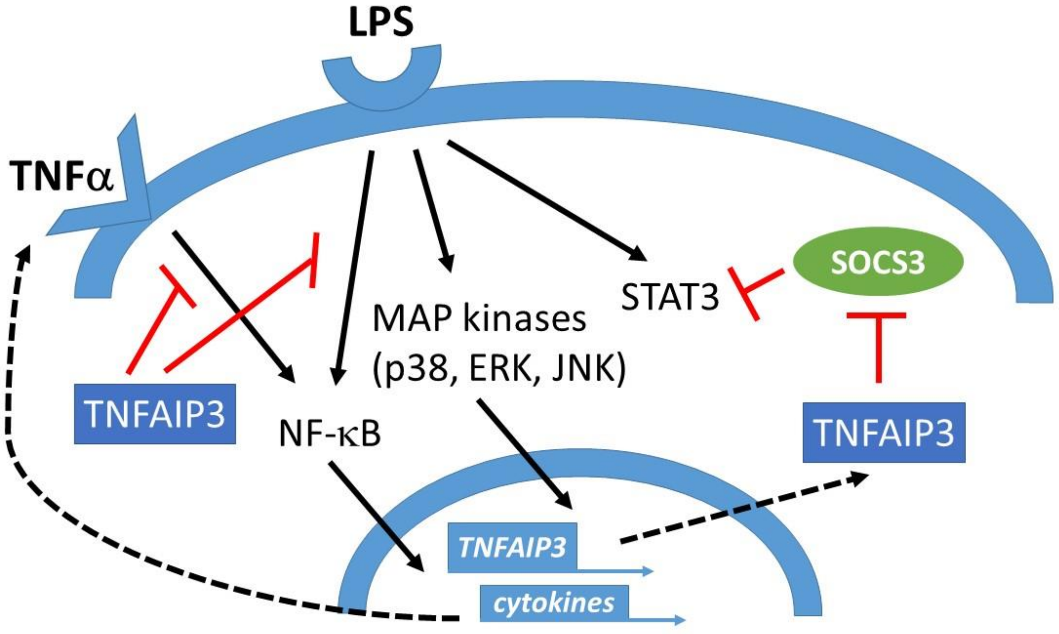

Figure S2: TNFAIP3 signaling diagram. Stimulation via inflammatory cytokines or pattern recognition receptor agonists (e.g. lipopolysaccharide or LPS) trigger the activation of signaling molecules such as MAP kinases and ultimately transcription factors such as NF-κB. NF-κB in turn induces the transcription of *TNFAIP3*. TNFAIP3 is an anti-inflammatory ubiquitin ligase/deubiquitinating enzyme that inhibits further LPS or cytokine signaling, thus putting the brakes on inflammation. TNFAIP3 also inhibits SOCS3, relieving SOCS3 downregulation of STAT3.

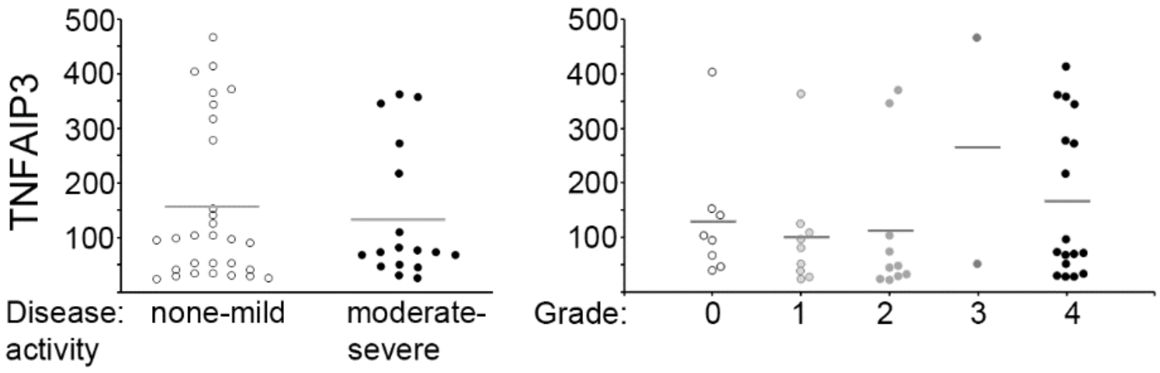

Figure S3 TNFAIP3 levels do not correlate with reported disease activity or sacroiliitis severity. A) Provider notes closest to time of sample collection were reviewed, and based on these notes, disease activity was described as none, mild, moderate or severe. TNFAIP3 levels for none-mild are in open circles and moderate to severe in closed circles. B) Sacroiliitis reports were reviewed and graded according to modified New York criteria as 0 (no sclerosis), 1 (sclerosis without mention of erosions), 2 (bilateral sclerosis with erosions), 3 (marked sclerosis with pronounced joint changes), or 4 (partial or full fusion). For both A) and B), gray lines denote means. Linear regression analysis and spearman's correlation did not reveal any significant relationships between sacroiliitis grade and TNFAIP3 levels.

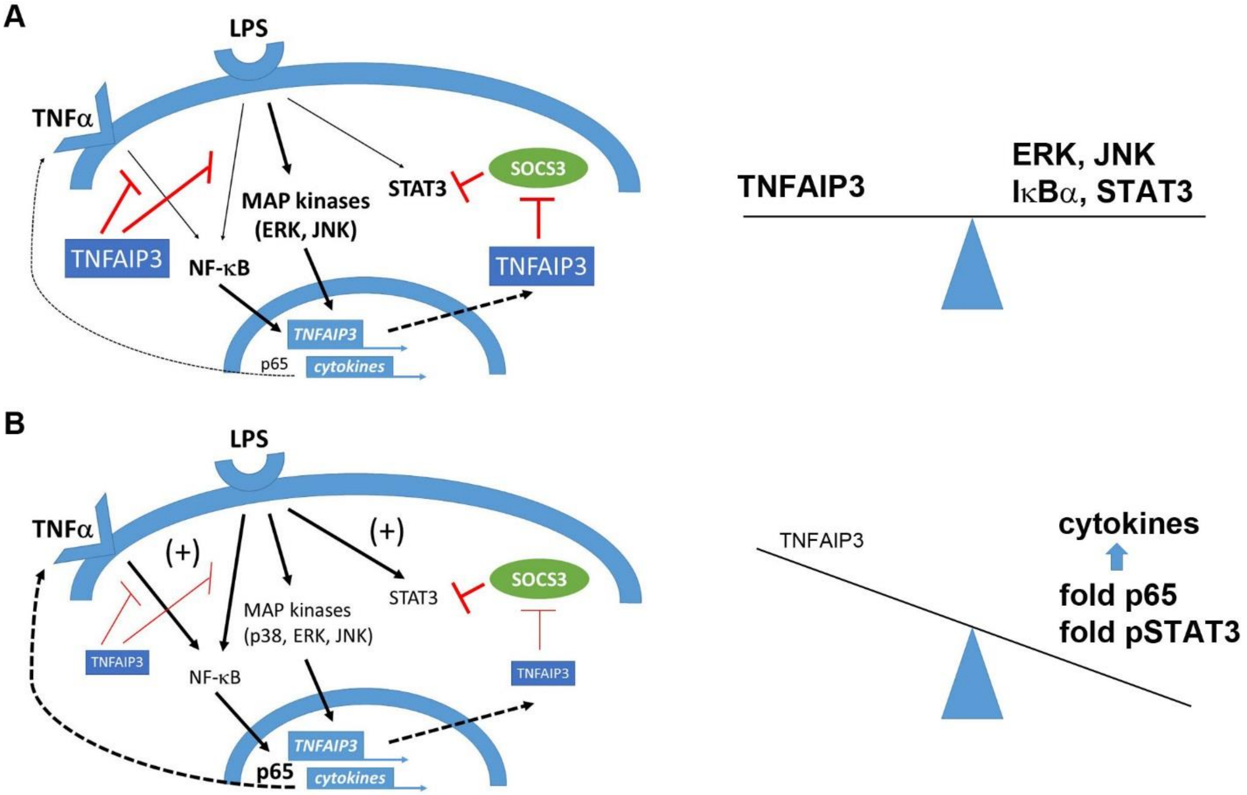

Figure S4: Summary of functional associations with TNFAIP3 and hypothesis for consequences. TNFAIP3 levels correlated positively with pIκBα, pERK, pJNK and pSTAT3, but negatively with fold-induction of pSTAT3, fold p65 and cytokine induction (TNF-α and IL-6). Hypothesis: under tonic or baseline elevated NF-κB or MAP kinase signaling, greater levels of TNFAIP3 keeps PRR or TNF-α induced inflammation in check (balanced). By inhibition of SOCS3, baseline pSTAT3 may also be higher. However, when TNFAIP3 levels are low, and baseline phosphorylation of these other pathways is similarly low, PRR stimulation triggers increased fold-induction of pSTAT3 and p65 (NF-κB), resulting in augmented cytokine induction.
